# Genome-wide comparative analysis of the valine glutamine motif containing genes in four *Ipomoea* species

**DOI:** 10.1186/s12870-023-04235-6

**Published:** 2023-04-22

**Authors:** Zengzhi Si, Lianjun Wang, Zhixin Ji, Yake Qiao, Kai Zhang, Jinling Han

**Affiliations:** 1 Hebei Key Laboratory of Crop Stress Biology, Hebei Normal University of Science and Technology, Qinhuangdao, 066000 China; 2 Institute of Food Corps, Hubei Academy of Agricultural Sciences, Wuhan, 430072 China

**Keywords:** *Ipomoea* species, *VQ* genes, Phylogenetic analysis, Chromosome location, Duplication analysis, *Cis*-regulatory elements, Expression patterns, Sweetpotato, Stresses response

## Abstract

**Background:**

Genes with valine glutamine (VQ) motifs play an essential role in plant growth, development, and resistance to biotic and abiotic stresses. However, little information on the *VQ* genes in sweetpotato and other *Ipomoea* species is available.

**Results:**

This study identified 55, 58, 50 and 47 *VQ* genes from sweetpotato (*I. batatas*), *I.triflida*, *I. triloba* and *I. nil*, respectively. The phylogenetic analysis revealed that the *VQ* genes formed eight clades (I–VII), and the members in the same group exhibited similar exon–intron structure and conserved motifs distribution. The distribution of the *VQ* genes among the chromosomes of *Ipomoea* species was disproportional, with no *VQ* genes mapped on a few of each species' chromosomes. Duplication analysis suggested that segmental duplication significantly contributes to their expansion in sweetpotato, *I.trifida*, and *I.triloba*, while the segmental and tandem duplication contributions were comparable in *I.nil*. *Cis*-regulatory elements involved in stress responses, such as W-box, TGACG-motif, CGTCA-motif, ABRE, ARE, MBS, TCA-elements, LTR, and WUN-motif, were detected in the promoter regions of the *VQ* genes. A total of 30 orthologous groups were detected by syntenic analysis of the *VQ* genes. Based on the analysis of RNA-seq datasets, it was found that the *VQ* genes are expressed distinctly among different tissues and hormone or stress treatments. A total of 40 sweetpotato differentially expressed genes (DEGs) refer to biotic (sweetpotato stem nematodes and *Ceratocystis fimbriata* pathogen infection) or abiotic (cold, salt and drought) stress treatments were detected. Moreover, *IbVQ8*, *IbVQ25* and *IbVQ44* responded to the five stress treatments and were selected for quantitative reverse-transcription polymerase chain reaction (qRT-PCR) analysis, and the results were consistent with the transcriptome analysis.

**Conclusions:**

Our study may provide new insights into the evolution of *VQ* genes in the four *Ipomoea* genomes and contribute to the future molecular breeding of sweetpotatoes.

**Supplementary Information:**

The online version contains supplementary material available at 10.1186/s12870-023-04235-6.

## Background

Plants are gradually exposed to various biotic and abiotic stresses during their life cycle. How to balance the relationship between growth and stresses to achieve the optimal utilization of energy is a challenge that plants often face. In the long-term evolution, plants have evolved elaborate mechanisms to respond to various external stimuli [1]. At the molecular level, reprogramming resistance-related genes in spatiotemporal expression is a virtual event for plants to adapt to adversity, and transcription factors (TFs) play an essential role in it. In most cases, TFs form complexes with transcription cofactors (TCs) through protein–protein interaction to achieve accurate and effective regulation of target genes [2].

Among the significant TCs, the valine-glutamine (VQ) motif-containing proteins work independently or with other TFs to regulate plant growth, developmental processes, and responses to biotic and abiotic stresses [3]. The name of the VQ protein originates from the conservative FxxxVQxhTG motif (VQ motif, h is a hydrophobic residue, and X represents any amino acid). It is reported that *VQ* genes play a vital role in plant growth differentiation, seed growth and development, and biotic or abiotic threats [3–6]. For example, the Arabidopsis *VQ* gene (*IKU1*) is involved in seed development [5]. *AtVQ14* was strongly expressed in the centrosome cells and endosperm and affects the seed size by adjusting the endosperm growth and development [7]. AtVQ21 protein interacts with the WRKY33 transcription factor to express the *PAD3* gene and enhance plant resistance to pathogens [5]. AtVQ9 is involved in the negative regulation of salt stress response by interacting with WRKY8 transcription factor, reducing the binding activity of the WRKY8 to DNA [6]. Recently, OsVQ14 and OsVQ 32, two rice VQ proteins, have been identified as MAPK cascade (OsMPKK6-OsMPK 4) signal components to adjust rice resistance to *Xanthomonas oryzae pv. oryzae (Xoo)* [8]; the *VQ1* gene of poplar grants the transgenic Arabidopsis salt tolerance and pathogen resistance via changing of hormone signal [9]; OsVQ25 protein can balance broad-spectrum disease resistance and plant growth by interacting with U-Box E3 ligase OsPUB73 and transcription factor OsWRKY53 [10].

*VQ* genes were first identified in Arabidopsis [11]. As many plant genome sequences have become available, the *VQ* gene family has since been identified in many plants. So far, 34, 39, 74, 18, 57, 61, 51, 27, 59, 118, and 113 *VQ* genes have been identified in *Arabidopsis thaliana* [12], *Oryza sativa* [13], *Glycine max* [14], *Vitis vinifera* [15], *Brassica rapa* [16], *Zea mays* [17], *Populus simonii* [18], *Eucalyptus grandis* [19], *Nicotiana tabacum* [20], *Brassica napus* [21], and *Triticum aestivum* [22], respectively. As talked about above, the difference in the number of *VQ* genes is approximately 2–6 times. The number of *VQ* genes varied greatly in the genomes of different species.

*Ipomoea* is the main genus of Convolvulaceae, including 600–700 species [23], which is distributed worldwide and is necessary for agriculture, animal husbandry and industry [23–25]. As the seventh key crop in the world, sweetpotato is an indispensable food and feed crop and a primary industrial raw material for energy plants [24, 26]. *I. trifida* and *I. triloba* are efficient plants for studying the genome evolution of *Ipomoea* due to their smaller genomes [27, 28]. As the closest wild diploid relatives of hexaploid sweetpotato [29], they are crucial for genetic improvement and relevant evolutionary analysis of sweetpotato. *I. nil* is planted as an ornamental plant for its diverse flower color patterns [25, 30]. It is generally used as a sticky wood mediated by sweetpotato grafting to induce a genetic variation of flowering and flowers [31]. However, there is no comparative analysis report on *VQ* genes in sweetpotato, *I. trifida*, *I. triloba*, and *I. nil*, and the evolutionary model of *VQ* genes in sweetpotato and its wild relatives is still unclear.

In this study, the genome-wide identification and expression analysis of the *VQ* gene was carried out for the four *Ipomoea* species: *I. batatas* (sweetpotato), *I. trifida*, *I. triloba*, and *I. nil*. To provide comprehensive information on this gene family, gene structure, conserved motifs, phylogenetic analysis, chromosomal distribution, duplication pattern, syntenic analysis, and Ka/Ks analysis of the identified *VQ* genes were conducted. After that, different RNA-seq datasets, referred to tissues and stresses, were used for expression analysis of these genes. Based on the results, 40 sweetpotato differentially expressed genes (DEGs) were identified and were further selected for quantitative reverse-transcription polymerase chain reaction (qRT-PCR) analysis. This study brings basic information for further research on the efficacy of *VQ* genes and lays a foundation for future molecular breeding of sweetpotato.

## Results

### Identification of the *VQ *genes in the four *Ipomoea* species

A total of 210 *VQ* genes were identified: 55 in *I. batata* (sweetpotato), 58 in *I. trifida*, 50 in *I. triloba*, and 47 in *I. nil*, which account for 0.07, 0.13, 0.11, and 0.11% of the genes of the genomes, respectively (Additional file 1: Table S1). These *VQ* genes were named from *IbVQ1* to *IbVQ55*, *ItfVQ1* to *ItfVQ58*, *ItbVQ1* to *ItbVQ50*, and *InVQ1* to *InVQ4*. Among the *VQ* genes in the four *Ipomoea* species, the average length of the protein sequences contains 215.81 amino acids (aa), ranging from 78 to 415 aa. The average length of the protein sequences in *I. nil* was the largest (225.85 aa, ranging from 97 to 415 aa), followed by *I. triloba* (220.08 aa, ranging from 93 to 386 aa), *I. trifida* (213.17 aa, ranging from 87 to 395 aa), and sweetpotato (206.15 aa, ranging from 78 to 383 aa). *VQ* genes in sweetpotato were predicted to have 1.56 exons on average, ranging from 1 to 5, which was much larger than the average number of exons in *I. nil* (1.43, ranging from 1 to 3), *I. triloba* (1.3, ranging from 1 to 3), and *I. trifida* (1.29, ranging from 1 to 4) (Additional file 1: Table S1).

### Phylogenetic analysis of the *Ipomoea VQ* genes

**Fig. 1:**
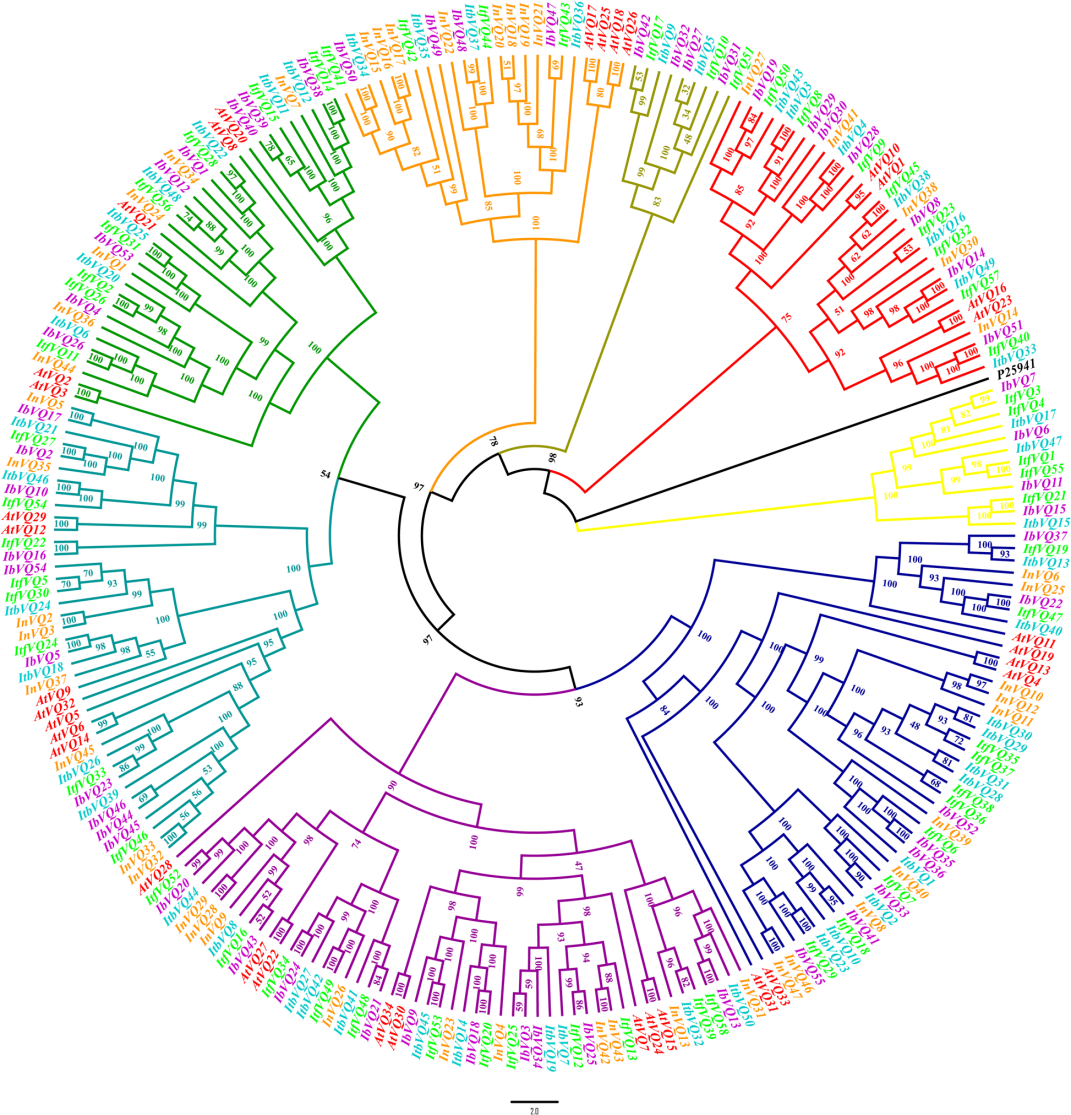
Phylogenetic tree of the *VQ* genes in Sweetpotato, *I. trifida*, *I. triloba*, *I. nil*, and Arabidopsis. The *VQ* gene names of *I. batatas*, *I. trifida*, *I. triloba*, *I. nil*, and *Arabidopsis* were colored with purple, green, cyan-blue, orange, and red, respectively. Red, orange, green, blue, cyan-blue, purple, yellow, and olive lines represent the phylogenetic group I, II, III, IV, V, VI, VII, and VIII, respectively. P25941 was used as outgroup (marked as blue)

For exploring the phylogenetic relationship of the *VQ* genes in *Ipomoea* species, a phylogenetic tree was constructed based on the alignment of the *Ipomoea* VQ protein sequences with the *Arabidopsis thaliana* VQs as references and *S. coelicolor* accession protein (P25941) as an outgroup (Fig. 1). The *Ipomoea VQ* genes were clustered into eight distinct groups: I-VIII. Each group, except for groups VII and VIII, contained Arabidopsis, sweetpotato, *I. trifida*, *I. triloba*, and *I. nil VQ* genes, suggesting that the characteristics of the *VQ* gene family appeared before these species’ differentiation. Of these eight groups, group VI was the largest (51 VQ proteins), followed by group IV (44 VQ proteins), V (39 VQ proteins), III (34 VQ proteins), I (31 VQ proteins), II (24 VQ proteins), VII (12 VQ proteins), and VIII (9 VQ proteins).

### Conserved motifs and structures of the *Ipomoea VQ* genes

**Fig. 2:**
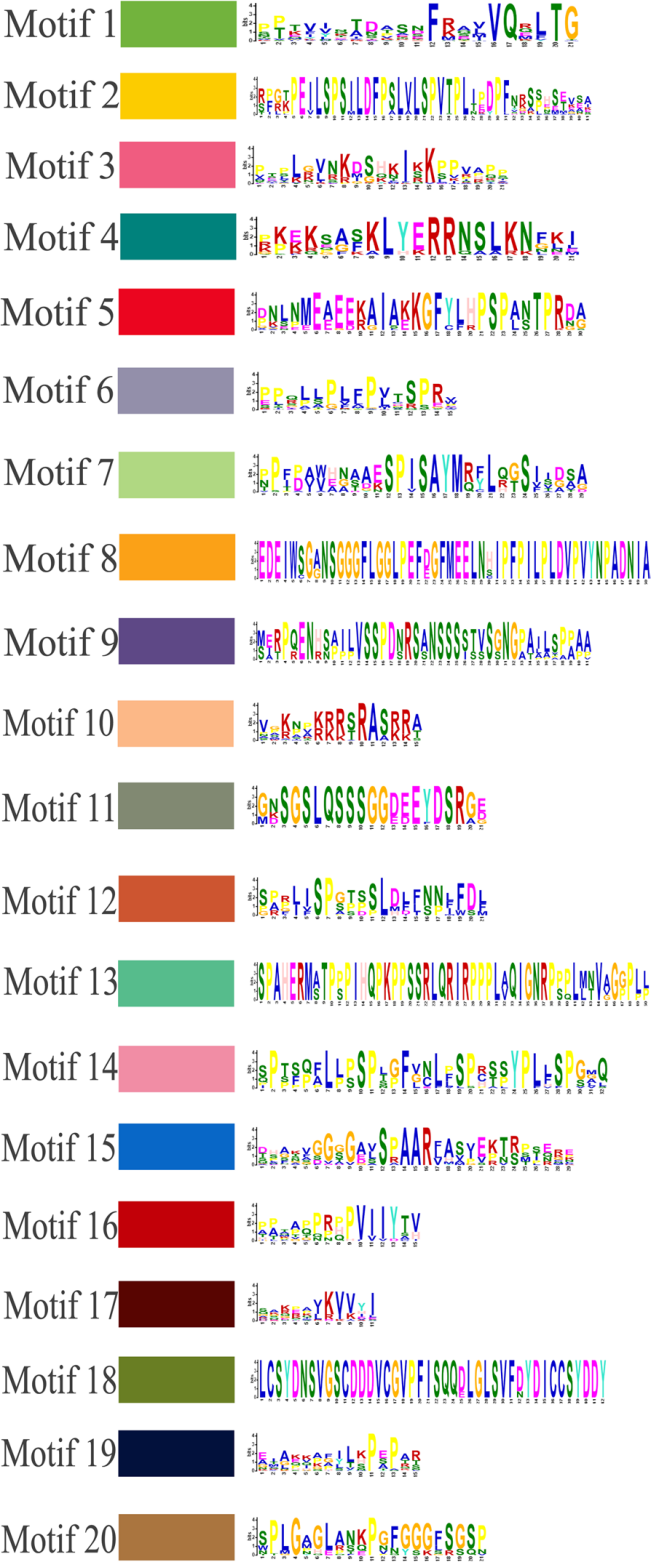
Conserved motifs of the *Ipomoea* VQ proteins. Each motif is marked with a specific color and a unique number (1–20)

**Fig. 3:**
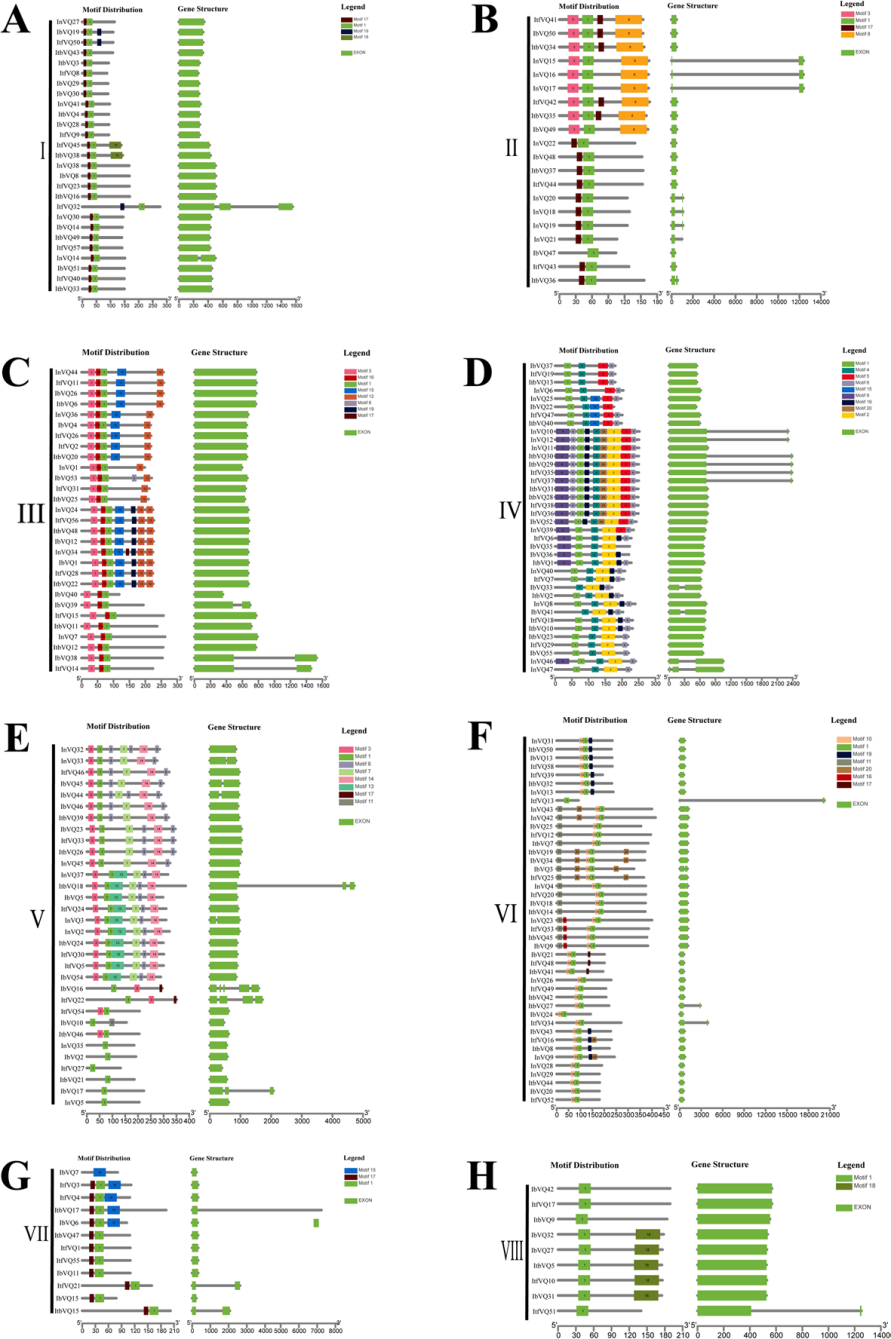
The motif distribution and exon–intron structures for *Ipomoea* VQ proteins of the eight phylogenetic groups. **A**-**H**: Group I-VIII

The conserved motifs of *Ipomoea VQ* protein were predicted using the MEME tool. A total of 20 motifs were detected (Fig. 2). Of these motifs, motif-1 (VQ-motif) aroused in nearly all of the *Ipomoea* VQ proteins (203 of 210) and was the most conserved (Fig. 2 and Fig. 3). It was followed by motif 3 (60 of 210), motif 6 (59 of 210), motif 17 (54 of 210), motif 10 (42 of 210), motif 19 (42 of 210) (Fig. 2 and Fig. 3). Most of the *Ipomoea* VQ genes contain only one exon and no intron (Fig. 3). The *Ipomoea VQ* genes from the same phylogenetic group tended to share the same conserved motifs (Fig. 3). VQ-motif was detected in all of the eight phylogenetic groups. Some motifs were seen only in one phylogenetic group. For instance, motif 8 only appeared in phylogenetic group II; motif 12 in phylogenetic group III; motif 2, motif 4, motif 5 and motif 9 in phylogenetic group IV; motif 7, motif 13 and motif 14 in phylogenetic group V, motif 10 in phylogenetic group VI (Fig. 3).

### Chromosomal location analysis of the *Ipomoea VQ* genes

**Fig. 4:**
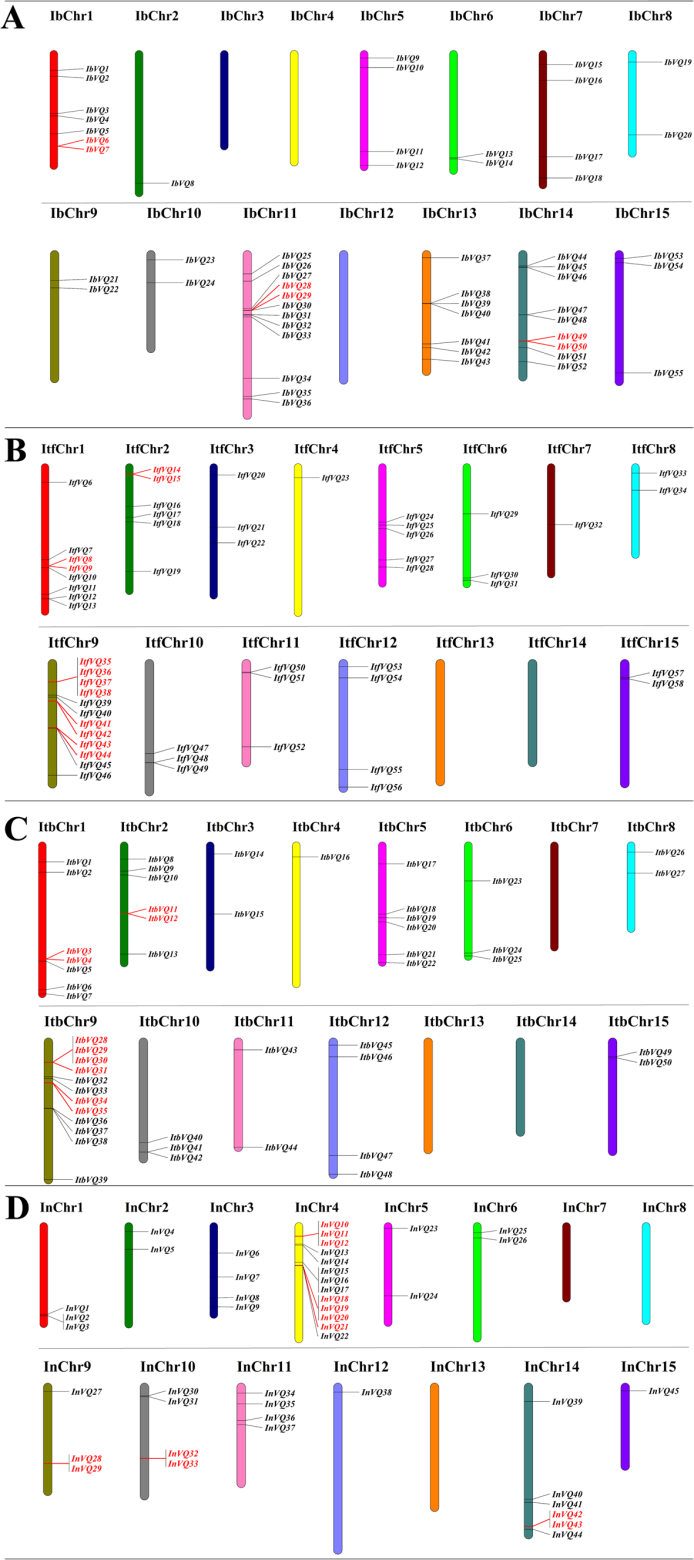
Distribution of *VQ* genes across the chromosomes of four *Ipomoea* species. (**A**) Sweetpotato; (**B**) *I. trifida*; (**C**) *I. triloba*; (**D**) *I. nil*. The red color indicates the tandemly duplicated *VQ* genes

Based on the physical location of individual *VQ* genes, 55, 53, 50, and 45 *VQ* genes were mapped throughout the 15 chromosomes of sweetpotato, *I. trifida*, *I. triloba*, and *I. nil*, respectively (Fig. 4 and Additional file 1: Table S1). The remaining *VQ* genes (5 genes in *I. trifida* and 2 genes in *I. nil*) were located on the other scaffolds that had not yet been linked to a chromosome. The distribution of sweetpotato *VQ* genes was disproportional across the 15 chromosomes. For example, there was 12, 9, 7 and 7 *VQ* genes mapping on chromosome 11, 14, 13, and 1 of sweetpotato, respectively, while no *VQ* genes mapped on sweetpotato chromosome 3, 4, and 12 at all (Fig. 4 A). This phenomenon, unbalanced distribution of *VQ* genes on chromosomes, was also found in *I. trifida*, *I. triloba*, and *I. nil* (Fig. 4 B-D).

### Duplication pattern analysis of the *Ipomoea VQ* genes

**Fig. 5:**
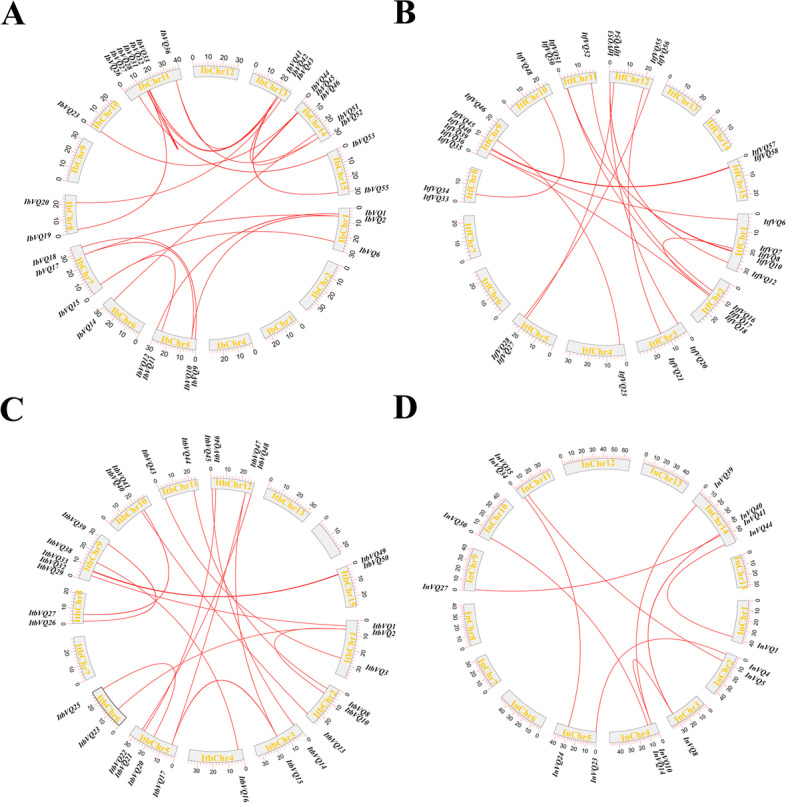
Collinear gene pairs for *VQ* genes on chromosomes of the four *Ipomoea* species. (**A**) *I. batatas*; (**B**) *I. trifida*; (**C**) *I. triloba*; (**D**) *I. nil*. The outer circle represents the haploid chromosomes (gray). Red lines show the collinear gene pairs for *VQ* genes

To better study the evolution of the *Ipomoea* VQ genes, their duplication pattern analysis was performed using MCScanX software. The total number of duplicated *VQ* gene pairs (both tandemly duplicated and segmentally duplicated ones) in sweetpotato (#27), *I. trifida* (#25), *I. triloba* (#25)was comparable and was more extensive than that in *I. nil* (#20) (Additional file 2: Table S2). Of these duplicated genes, a total of 3, 7, 6, and 11 gene pairs in sweetpotato, *I. trifida*, *I. triloba*, and *I. nil* were detected as tandemly duplicated genes, respectively; 24, 18, 19, and 9 *VQ* gene pairs were identified as segmentally duplicated genes (Fig. 4, Fig. 5 and Additional file 2: Table S2). In the genome of sweetpotato, *I. trifida*, and *I. triloba*, segmental duplications were more than tandem duplications, suggesting that segmental duplications were predominant in the expansion of the four *Ipomoea* species *VQ* genes. While in *I. nil*, the contributions of the two type duplication patterns were comparable (Additional file 2: Table S2). The duplications occurred in all of the eight phylogenetic groups, with group IV harboring the most significant number of duplicated genes, followed the group I, V, VI, III, II, VII and VIII (Additional file 2: Table S2). The duplications generally appeared within the phylogenetic group in the four *Ipomoea* species. For instance, *IbVQ2* and *IbVQ10* were detected as a duplicated gene pair, and both of them belong to phylogenetic group V, while a few duplicated gene pairs in *I. nil* (especially in segmental duplications) belonged to the group I, III, and IV were exceptions (Additional file 2: Table S2).

### Syntenic analysis of *VQ *genes in the genomes of the four *ipomoea* species

**Fig. 6:**
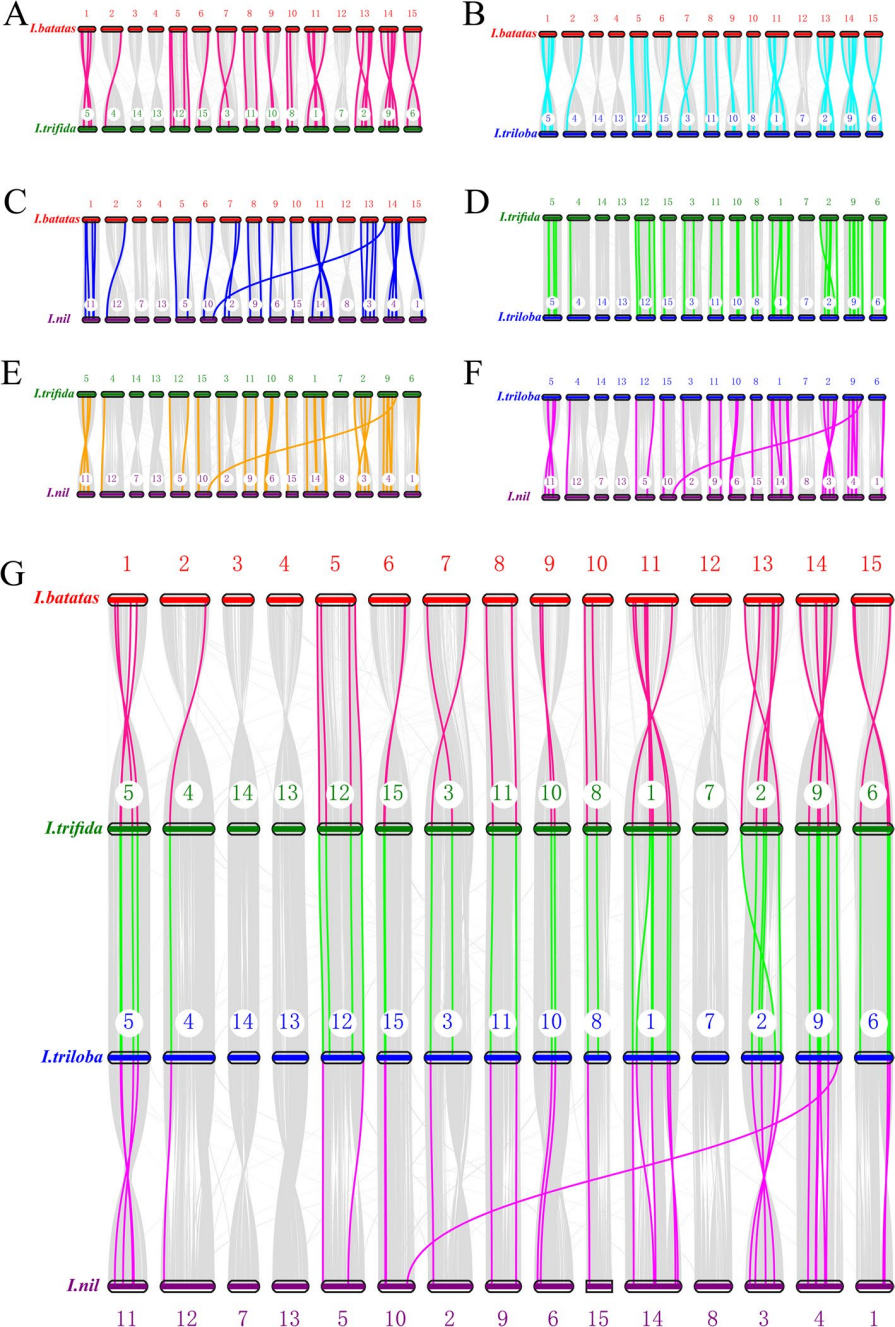
Synteny analyse of the *VQ* genes between *Ipomoea* species. (**A**) Sweetpotato and *I. trifida*. (**B**) Sweetpotato and *I. triloba*. (**C**) Sweetpotato and *I. nil*. (**D**) *I. trifida* and *I.triloba*. (**E**) *I. trifida* and *I. nil*. (**F**) *I.triloba* and *I.nil*. (**G**) Schematic representation of syntenic genes among sweetpotato, *I. trifida*, *I. triloba*, and *I. nil*. The chromosomes of sweetpotato, *I. trifida*, *I. triloba*, and *I. nil* were colored with red, green, blue, and purple, respectively. Gray lines connect matched gene pairs, with *VQ* gene pairs highlighted in pink, cyan-blue, blue, green, orange, and purple

Synteny analysis showed that there were 33, 34, 29, 25, 24, and 24 syntenic blocks of *VQ* genes containing 44, 45, 33, 45, 33 and 33 collinear pairs between *I. batatas* and *I. trifida*, *I. batatas* and *I. triloba*, *I. batatas* and *I. nil*, *I. trifida* and *I. triloba*, *I. trifida* and *I. nil*, *I. triloba* and *I. nil* groups, respectively (Fig. 6 and Additional file 3: Table S3). The collinear pairs occurred in all eight phylogenetic groups, and each team consisted of genes from the same phylogenetic group, with a few couples between *I. trifida* and *I. nil* were exceptions (Table S4). A total of 120 *VQ* orthologous genes (30 from *I. batatas*, 30 from *I. trifida*, 30 from *I. triloba*, and 30 from *I. nil*) formed 30 orthologous groups, any two of the *VQ* genes in each group can form synteny gene pairs (Fig. 6 and Additional file 4: Table S4).

### Ka/Ks analysis of duplicated and syntenic *Ipomoea VQ* genes

To detect whether duplicated and syntenic *VQ* genes are under positive selection, Ka/Ks analysis was performed (Additional file 5: Table S5). Most duplicated and syntenic *VQ* genes have a Ka/Ks ratio of less than one, suggesting that most of these genes underwent purifying evolutionary selection. Only one pair of tandem duplicated VQ genes (*InVQ20* and *InVQ21*) possessed a higher Ka/Ks ratio, i.e., 1.14 in *I. nil*. There were three and one syntenic *VQ* gene pairs that kept Ka/Ks ratio greater than 1 between *I. batatas* and *I. trifida* (*IbVQ20* and *ItfVQ52*, 1.08; *IbVQ30* and *ItfVQ8*, 1.31; *IbVQ11* and *ItfVQ55*, 1.40), and *I. trifida* and *I. triloba* (*ItfVQ55* and *ItbVQ47*, 1.41), respectively (Additional file 5: Table S5). These results suggest that most duplicated and syntenic *VQ* genes were subjected to purifying selection inside the duplicated genomic elements during speciation. In contrast, fewer such genes were subjected to positive selection.

### Stress-related regulatory elements analysis in promoter regions of the *Ipomoea VQ* genes

**Fig. 7:**
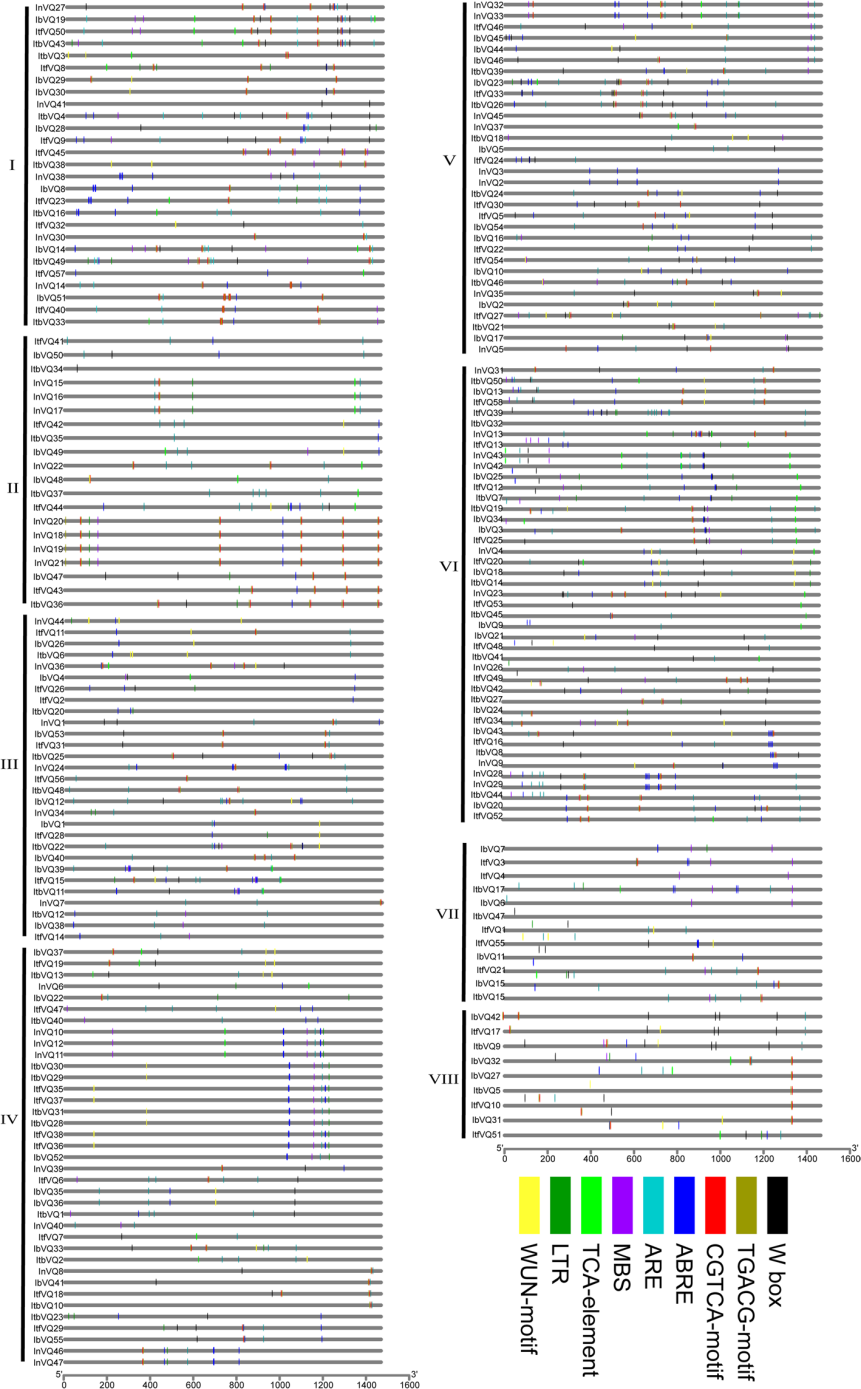
Stress-related regulatory elements distribution in promoter sequences of *Ipomoea VQ* genes. I-VIII refer to the eight phylogenetic groups. Different elements marked with different colors

The 1,500 bp upstream regulatory regions of all *Ipomoea VQ* genes were used to explore stress-related regulatory elements. Various elements were detected. In this present investigation, W-box, TGACG-motif, CGTCA-motif, ABRE, ARE, MBS, TCA-elements, LTR, and WUN-motif were calculated (Fig. 7). A total of 1740 elements were detected, the largest one was ABRE (#362), followed by ARE (#326), TGACG-motif (#241), CGTCA-motif (#241), W-box (#204), MBS (#117), WUN-motif (#98), LTR (#79), and TCA-element (#72). A few *VQ* genes phylogenetically clustered together trended to have somewhat similar elements distribution, for example, *IbVQ52*, *ItfVQ36*, *ItfVQ38*, *ItbVQ28*, *ItbVQ31*, *ItfVQ37*, *ItfVQ35*, *ItbVQ29*, and *ItbVQ30* were clustered together in group V, and they all had a similar ABRE-MBS-ARE distribution in similar regions of their promoter sequences (Fig. 7). However, the majority of the *VQ* genes have different elements distribution in their promoter sequences (Fig. 7). It was also found that the predominant elements in each phylogenetic group were other, for instance, CGTCA-motif and TGACG-motif were most elements in group I and II, ABRE was the most elements in group III, IV, V, VI and VII, W-box was the most elements in group VIII (Additional file 6: Table S6).

### Expression patterns of the *VQ *genes in the four* Ipomoea* species

**Fig. 8:**
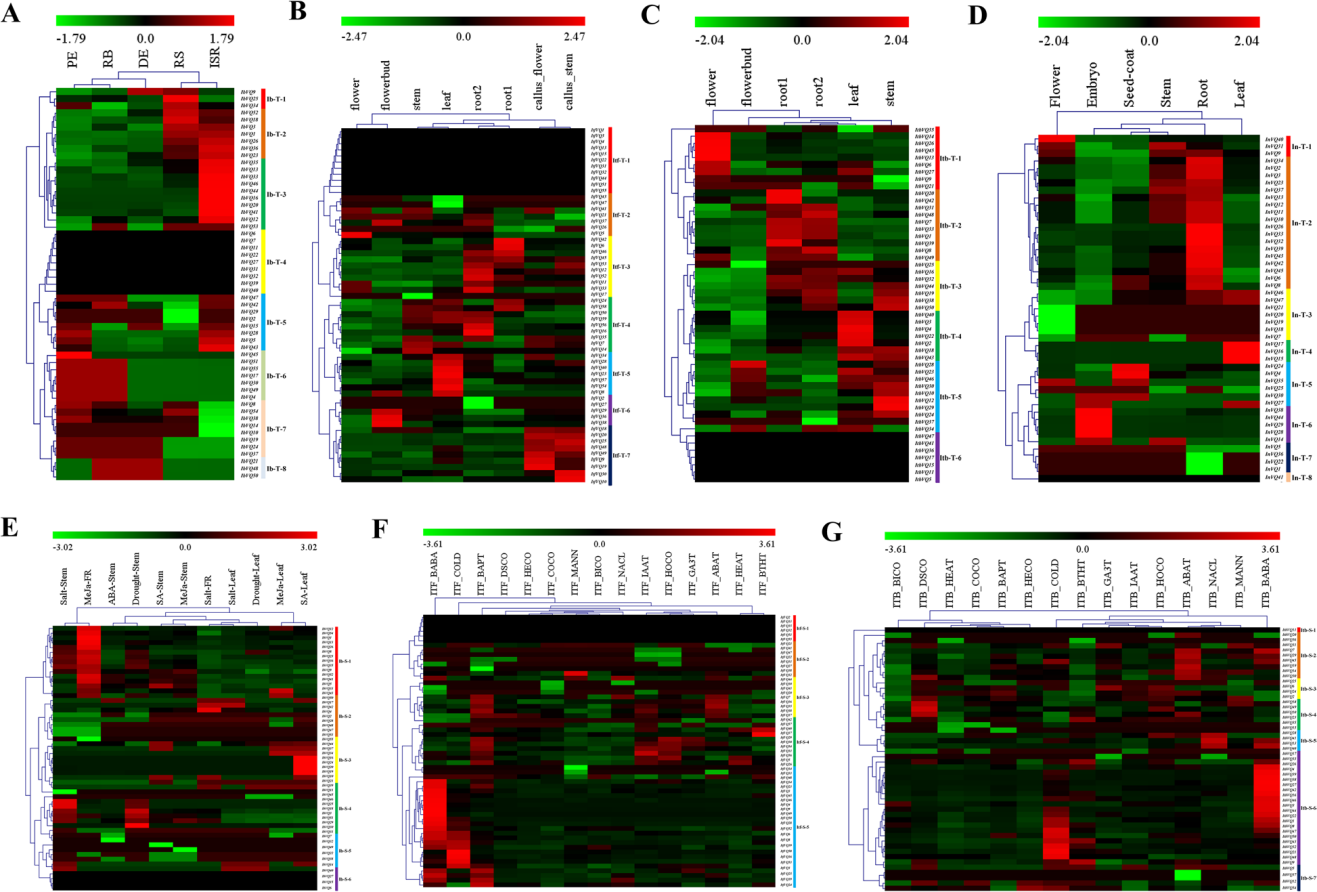
Genes expression profiles of *VQ* genes in sweetpotato, *I. trifida*, *I. triloba*, and *I. nil*. **A**-**D** *VQ* gene profiles in tissues of sweetpotato, *I. trifida*, *I. triloba*, and *I. nil*, respectively; **E**–**G** Expression profiles of *VQ* genes in sweetpotato, *I. trifida* and *I. triloba* in response to stress treatments, respectively

By analyzing the public RNA-seq datasets, the expression profiles of *Ipomoea VQ* genes in different tissues and stress treatments were obtained (Fig. 8). In various tissues, 8, 7, 6 and 8 expression pattern groups, named from Ib-T-1 to Ib-T-8, from Itf-T-1 to Itf-T-7, from Itb-T-1 to Itb-T-6, and from In-T-1 to In-T-8, were acquired in sweetpotato, *I. trifida*, *I. triloba*, and *I. nil*, respectively (Fig. 8). The *VQ* genes in group Ib-T-4, Itf-T-1, Itb-T-6 and In-T-8 had no regulation in all of investigated tissues in the four *Ipomoea* species. The *VQ* genes in other group were up- or down-regulated in various tissues, and trended to somewhat group-specially regulate (Fig. 8). Take sweetpotato for example, the *VQ* genes in group Ib-T-3 were upregulated in initiative storage root (ISR), and downregulated in fibrous root (FR), distal end (DE), proximal end (PE), root body (RB), and root stalk (RS); while the *VQ* genes in group Ib-T-6 were upregulated in PE and RB, and downregulated in other tissues (Fig. 8). In different stress treatments, 6, 5 and 8 expression pattern groups, named from Ib-S-1 to Ib-S-6, from Itf-S-1 to Itf-S-5, and from Itb-S-1 to Itb-S-8, were acquired in sweetpotato, *I. trifida*, and *I. triloba*, respectively (Fig. 8). The *VQ* genes in sweetpotato showed somewhat tissue-special regulation under the same stress treatment, for instance, under methyl jasmonate (MeJa) treatment, the *VQ* genes in group Ib-S-1 were mainly upregulated in fibrous root (MeJa-FR), while they were primarily down-regulated in leaf (MeJa-leaf) (Fig. 8). The *VQ* genes in group Itf-S-5 of *I. trifida* and in group Itb-S-6 of *I. triloba* were mainly upregulated in beta-aminobutyric acid biotic stress experiment (ITF_BABA and ITB_BABA), and more than a half of them were upregulated in cold stress at 10/°C day/night experiment (ITF_COLD and ITB_COLD) as well, while the major of them were mainly downregulated under the other stress treatments (Fig. 8).

### Transcriptome analysis of the *VQ* genes response to biotic and abiotic stresses in sweetpotato

**Fig. 9:**
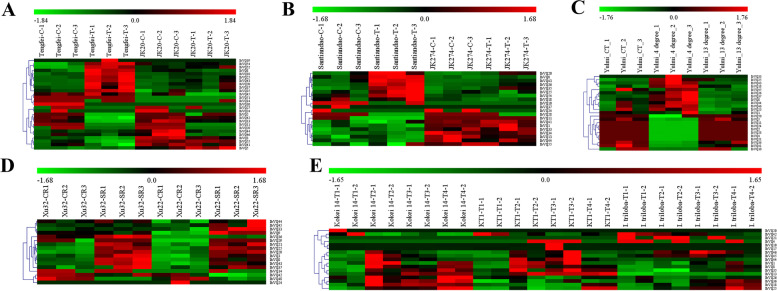
Heatmap of the expression profiles of sweetpotato differentially expressed genes (DEGs) in response to biotic and abiotic stresses. **A** DEGs in “Tengfei” and “JK20” under control and sweetpotato stem nematodes inoculation. C, control; T, treatment. **B** DEGs in “Santiandao” and “JK274” under control and *Ceratocystis fimbriata* inoculation. C, control; T, treatment. **C** DEGs in “Yulmi” under control (CT), 4 °C and 13 °C. **D** DEGs in “Xu 32” and “Xu 22” under control and salt treatments. CR, control; SR, treatment. **E** DEGs in “Kokei 14”, “KT 1” and “*I. triloba*” under control and drought treatments

To explore the *VQ* genes’ response to biotic and abiotic stresses in sweetpotato, five RNA-seq datasets, referred to as sweetpotato stem nematode resistance, *Ceratocystis fimbriata* pathogen resistance, cold tolerance, salt tolerance and drought tolerance, were analyzed. A total of 27, 20, 23, 17 and 17 differentially expressed genes (DEGs) in the analysis of sweetpotato stem nematode resistance, *Ceratocystis fimbriata* pathogen resistance, cold tolerance, salt tolerance and drought tolerance were detected, respectively (Fig. 9 and Additional file 7: Table S7). The total number of DEGs related to either of the above stress treatment was 40, and three of them (*IbVQ8*, *IbVQ25* and *IbVQ44*) were considered as DEGs in all of the above stress treatments (Fig. 9 and Additional file 7: Table S7). Of the 40 DEGs, the number belonged to phylogenetic group VI was the largest (#10), followed by group V (#9), IV (#7), I (#6), III (#3), II (#2), VIII (#2) and VII (#1) (Additional file 7: Table S7).

### Expression analysis of sweetpotato *VQ* genes by quantitative reverse-transcription polymerase chain reaction (qRT-PCR)

**Fig. 10:**
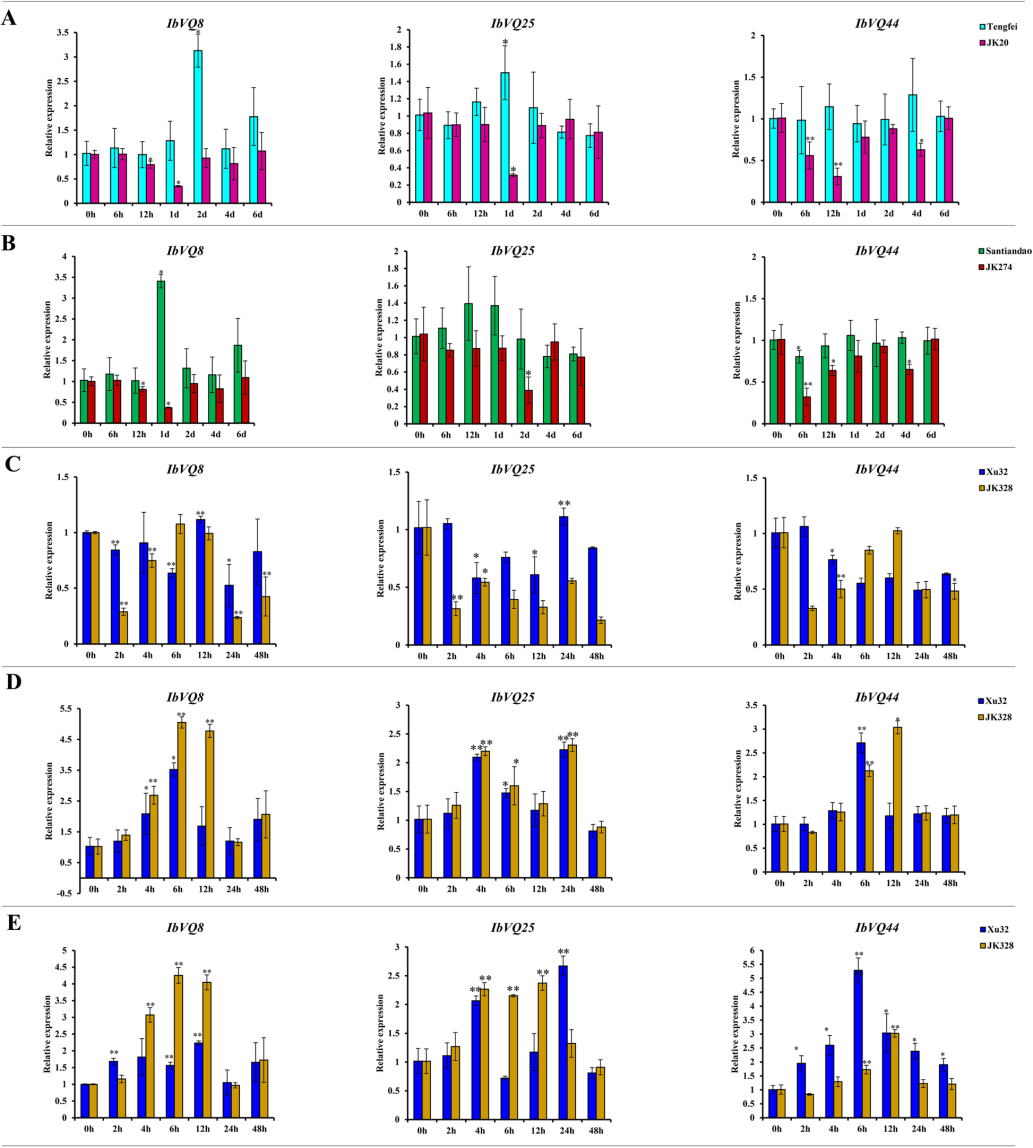
Expression analysis of *IbVQ8*, *IbVQ25* and *IbVQ44* in sweetpotato cultivars or lines. **A** Relative expression levels in storage roots after different times of sweetpotato stem nematode infection. **B** Relative expression levels after different times of *Ceratocystis fimbriata* infection. **C** Relative expression levels in leaves after different times of cold (16 °C) treatments. **D** Relative expression levels in leaves after different times of salt (86 mM NaCl) treatments. **E** Relative expression levels in leaves after different times of drought (30% PEG 6000) treatments. The significance of expression levels compared with control were denoted as ∗  < 0.05, ∗  ∗  < 0.01. h, hours; d, day(s)

Based on transcriptome results, *IbVQ8*, *IbVQ25* and *IbVQ44* were selected for further analysis using qRT-PCR. Compared with the control condition (0 h), the transcripts of *IbVQ8*, *IbVQ25* and *IbVQ44* in Tengfei (susceptible cultivar) were upregulated after sweetpotato stem nematode infection treatment, and increase to peak at 2 days with 3.13, 1 day with 1.50, and 4 days with 1.28, respectively; while the transcripts of them in JK20 (resistant line) were downregulated after infection, and decreased to valley at 1 day with 0.35, 1 day with 0.31, and 12 h with 0.31, respectively (Fig. 10). The transcripts of *IbVQ8* and *IbVQ25* in Santiandao (susceptible cultivar) were upregulated after *Ceratocystis fimbriata* inoculation compared with the control condition (0 h), and increase to peak at 1 day with 3.41, and 12 h with 1.39, respectively, and no obvious increasing or decreasing was found in the transcripts of *IbVQ44*; while the transcripts of them in JK274 (resistant line) were downregulated after infection, and dropped to valley at 1 day after infection with 0.36, 2 day with 0.39, and 6 h with 0.32, respectively (Fig. 10). Under cold treatment, compared with the control condition (0 h), the transcripts of *IbVQ8* in Xu 32 (susceptible cultivar) were slightly decreased at 2 h, slightly increased at 12 h, then reduced again at 24 h, while the transcripts of *IbVQ8* in JK328 (resistant line) were mainly downregulated, and decreased to valley at 1 day after cold treatment; the transcripts of *IbVQ25* and *IbVQ44* in Xu 32 had no significant regulation compared to the control, while the transcripts of them in JK328 were all downregulated (Fig. 10). Under salt and drought treatments, the transcripts of *IbVQ8*, *IbVQ25* and *IbVQ44* were upregulated in both Xu32 and JK328, the expression trend of each gene in Xu32 and JK328 was similar, and the expression level of each gene in Xu32 was usually higher than that in JK328 (Fig. 10).

## Discussion

The plant-specific *VQ* genes play essential roles in plant growth and development [5, 32–34], and biotic and abiotic stress resistance [6, 12, 13]. Given the importance of the *VQ* genes, genome-wide surveys of them have been performed in various species, such as Arabidopsis [12], *Oryza sativa* [13], *Zea mays* [17], and *Triticum aestivum* [22]. The *Ipomoea*, with great value in industry and agriculture, is the largest genus in Convolvulaceae [23, 25, 26]. However, no comprehensive and systematic research has been conducted on the *VQ* genes in the sweetpotato and other *Ipomoea* species.

To date, the genome sequences of several *Ipomoea* species were released, such as sweetpotato [35], *I. trifida* [36], *I. triloba* [36], *I. nil* [37], *I. cairica* [38] and *I. aquatic* [39]. Of these sequences species, sweetpotato is the seventh most important crop and the only staple crop in the genus *Ipomoea* that is widely cultivated and consumed worldwide. *I. trifida* and *I. triloba* were considered to be the closest extant relatives of the cultivated sweetpotato [29], and *I. nil* was once used as a reference to generate the sweetpotato genome [38]. It is generally used as a sticky wood mediated by sweetpotato grafting to induce a genetic variation of flowering and flowers [25, 30]. *I. cairica* and *I. aquatic* were close to each other while diverged from the other *Ipomoea* species.It was reported that *I. cairica* and *I. aquatica* diverged from each other 8.1 million years ago (MYA) and they diverged from the other *Ipomoea* species 9.8 MYA [38, 39]. Focus on excavating the *VQ* genes that could directly contribute to the molecular breeding of sweetpotatoes shortly, the comprehensive analysis of the *VQ* gene family has been conducted on sweetpotato, *I. trifida*, *I. triloba*, and *I. nil*. The results provided new insights and valuable information for *Ipomoea VQ* gene evolution and plant resistance breeding.

This study identified a total of 55, 58, 50, and 47 *VQ* genes from the genome of sweetpotato, *I. trifida*, *I. triloba*, and *I. nil*. The number of *VQ* genes slightly varied among the four *Ipomoea* species. Considering the whole gene numbers of each species, the proportion of *VQ* genes in *I. trifida*, *I. triloba* and *I. nil* was comparable (0.13%, 0.11% and 0.11%). It was higher than that in sweetpotato (0.07%). The results suggested that the number of *VQ* gene family members may not have an absolute correlation with genome size [40]. Such a phenomenon has also been reported in other species. The number of *VQ* genes is approximately 2–6 times different among species. For instance, the number of *VQ* genes in Arabidopsis was 34 (0.13%) [12, 41], in *Vitis vinifera* was 18 (0.06%) [14, 42], in *Triticum aestivum* was 118 (0.09%) [22, 43].

Our phylogenetic analysis of the *VQ* in the four *Ipomoea* species and Arabidopsis revealed eight independent groups: I to VIII. The *VQ* genes of the five species (Arabidopsis, sweetpotato, *I. trifida*, *I. triloba*, and *I. nil*) were distributed in each group of I to VI. The number of group members differed among species, indicating that the *VQ* genes have developed in multiple directions throughout evolution [44]. The *VQ* genes in Arabidopsis and *I. nil* tended to formatted species-special clusters, especially for the *VQ* genes in Arabidopsis. In contrast, the *VQ* genes in sweetpotato, *I. trifida*, and *I. triloba* were interspersed and clustered. Moreover, groups VII and VIII only contained *VQ* genes of sweetpotato, *I. trifida* and *I. triloba*. These results indicate that the sweetpotato VQ genes were more similar to *I. trifida* and *I. triloba* than *I.nil* and Arabidopsis. The results were consistent with the evolutionary relationship, as *I. trifida* and *I. triloba* were demonstrated as wild ancestors of the sweetpotato [45].

Based on gene structure analysis, most *Ipomoea VQ* genes have only one exon, i.e., no intron. The intron-free phenomenon of *VQ* genes has been reported in other species, such as *Arabidopsis thaliana* [12], *Oryza sativa* [13], *Zea mays* [17], poplar [18], sugarcane [40] and wheat [44]. This phenomenon could be because plant *VQ* genes have lost many introns during their evolutionary history [18]. The members of the same phylogenetic group tended to share similar conserved motifs, and some conserved motifs were only in a particular phylogenetic group. Previous studies have also reported this phenomenon in other species [18, 40, 44].

The gene duplication events, segmental and tandem duplication, played essential roles in gene family expansion and distribution of genes in plants [46–48]. Segmental duplications of multiple genes occurred through polyploidy followed by chromosome rearrangements. Tandem duplications arose within the same or neighboring intergenic regions [48]. In this study, the distribution of *Ipomoea VQ* genes was disproportional across the 15 chromosomes. Segmental duplication was predominant in sweetpotato, *I. trifida*, and *I. triloba*, while in *I. nil*, the contributions of the two types of duplication patterns were comparable. High segmental and low tandem duplication ratios were detected in the *VQ* genes of apple [32], *Brassica napus* [49], and *Cucurbita pepo* [50]. In sunflower (*Helianthus annuus* L.), the number of segmentally duplicated genes and tandemly duplicated ones was the same, a segment gene pair (*HaVQ*5-*HaVQ*20) was detected on chromosomes 4 and 17, and a tandem gene cluster (*HaVQ14*-*HaVQ15*) was localized to chromosome 12 [51].

This study obtained 30 orthologous groups of 120 *VQ* orthologous genes (30 from *I. batatas*, 30 from *I. trifida*, 30 from *I. triloba*, and 30 from *I. nil*). Synteny analysis of *VQ* genes in the four *Ipomoea* species showed strong collinearity even though the chromosomal rearrangements or gene duplication occurred between them after being divergent from their common ancestor [45, 52, 53]. To better understand the evolutionary characters of duplicated and syntenic *VQ* gene pairs of the four *Ipomoea* species, Ka/Ks analysis of them was calculated. The results showed that most *VQ* gene pairs had a Ka/Ks ratio less than 1, and a few harbored a Ka/Ks ratio larger than 1. These results suggest that the duplicated and syntenic *VQ* genes mainly undergo purifying (negative) selection within genome duplication and speciation, and a tiny portion shows a positive selection [54].

Regulatory elements are particular DNA sequences with transcriptional regulation functions in the same DNA molecule, and their analysis may improve our fundamental understanding of gene regulation [55, 56]. As expected, abundant cis-regulatory elements involved in biotic and abiotic responses were detected in the promoters of *Ipomoea* VQ genes, such as TGACG-motif, CGTCA-motif, W-box, ABRE, ARE, MBS, TCA-elements, LTR, and WUN-motif. The abundance of stress-related regulatory elements might be why detected a large proportion of stress responded to *VQ* genes in expression analysis.

Previous studies reported that *VQ* genes were not only involved in regulating plant growth and development [5, 32–34] but also in responses to biotic and abiotic stress [6, 12, 13]. The expression patterns of *VQ* genes were analyzed using public RNA-seq datasets in the present study. Various expression patterns were obtained (Fig. 8). As expected, the *Ipomoea* VQ genes behaved differently among various tissues of the investigated species and in different stress treatments.

Two RNA-seq datasets referred to biotic stresses (sweetpotato stem nematode and *Ceratocystis fimbriata* pathogen resistance) and three referred to abiotic stresses (cold, salt and drought) were selected to analyze, and 40 (72.7%) *VQ* DEGs were detected finally (Fig. 9). The *VQ* DEGs were up- or down-regulated under different stress treatments in sweetpotato, and trend to show other regulation between susceptible and resistant cultivars (lines). Based on these, three sweetpotato VQ genes (*IbVQ8*, *IbVQ25* and *IbVQ44*) were further analyzed using qRT-PCR, and the results were consistent with the RNA-seq analysis. *IbVQ8*, *IbVQ25* and *IbVQ44* were induced to upregulate in susceptible cultivars while downregulated in resistant lines under sweetpotato stem nematode and *Ceratocystis fimbriata* pathogen infection; they were unregulated or slightly upregulated in susceptible cultivars while also downregulated in resistant lines under cold treatments; they were induced to upregulated in both susceptible cultivars and resistant lines under both salt and drought treatments (Fig. 10). It was noteworthy that some upregulated *VQ* genes might harm stress resistance, i.e., stress could induce the expression of *VQ* genes, but overexpression them may make plants hypersensitive to stress. This phenomenon has been reported by previous studies, such as *AtVQ9* and *AtVQ15*, which were all introduced by salt stress. In contrast, their overexpression lines showed an increased sensitivity to salt stress, and the antisense lines were significantly more tolerant of these stresses [4, 16, 18, 57].

## Conclusions

This study has comprehensively analyzed the *VQ* genes of four *Ipomoea* species: sweetpotato (*I. batatas*), *I. trifida*, *I. triloba*, and *I. nil*. We identified 55, 58, 50 and 47 *VQ* genes in sweetpotato (*I. batatas*), *I. trifida*, *I. triloba* and *I. nil*, respectively. Based on phylogenetic analysis, the *VQ* genes were classed into eight monophyletic clades (I–VII). We have analyzed conversed motifs, gene structure, and disproportional chromosome distribution of *VQ* genes. Segmental duplication significantly contributes to the expansion of the *VQ* gene family in the four *Ipomoea* species, and we have identified 30 orthologous groups among the four *Ipomoea* species. We acquired the expression patterns of *VQ* genes and 40 sweetpotato differentially expressed genes (DEGs) referring to different biotic or abiotic stress. Moreover, three DEGs (*IbVQ8*, *IbVQ25* and *IbVQ44*) were further selected for qRT-PCR analysis, and the results were consistent with the transcriptome analysis. These results provide valuable information to understand the *Ipomoea VQ* genes and help determine candidate genes for molecular-assisted sweetpotato breeding.

## Methods

### Identification of* VQ* genes in four *ipomoea* species

The whole genome sequences and annotated gene model of the four *Ipomoea* species were obtained from the open access databases: sweetpotato from the *Ipomoea* Genome Hub (https://www.sweetpotao.com/download_genome.html) (version 3), *I. trifida* (ver. 3) and *I. triloba* (ver. 3) from GenBank BioProject (accessions numbers PRJNA428214 and PRJNA428241), and *I. nil* (ver. 1.2) also from GenBank BioProject (accession numbers BDFN01000001- BDFN01003416). HMMsearch (ver. 3.1b2) with default parameters is applied to search the VQ domain (Pfam accession number: PF05678) of all the protein sequences. At the same time, the extended amino acid sequence of the VQ domain was used as a query to search against all the protein sequences using the BLASTP program (ver. 2.2.28 +) (evalue 1e-10). Protein sequences obtained by HMMsearch and BLAST were merged and removed redundant ones. To further confirm the existence of the VQ domain, checked the candidate VQ proteins with HMMscan against the Pfam-A database by setting the E-value up to 0.0001.

### Identification of conserved motifs of the *VQ* genes

In order to study the diversity of the structural motifs of the *VQ* genes that have been detected, the motif analysis of the protein sequence is carried out using the online MEME SUITE (https://meme-suite.org/meme/) (ver. 5.5.3). The maximum number of motifs was designed to identify 20 motifs and the site distribution was set as any, while other parameters were set as default [58].

### Sequence alignment and phylogenetic analysis of *VQ* genes

The full lengths of the identified VQ proteins were first aligned with Clustal Omega [59, 60]. The obtained aligned sequences were summited to IQ-TREE (ver. 2.1.3) for phylogenetic analysis using the maximum likelihood approach [61]. Based on the analysis of ModelFinder (ver. 2.0) [62], the best-fit model VT + F + R4 was chosen. The branch support values were calculated using SH-aLRT and UFBoot2 with 1,000 bootstrap replicates [63], and *Streptomyces coelicolor* accession P25941 was set as an outgroup [64]. After that, the obtained phylogenetic tree was summited in Figtree (ver.1.4.3) for visual improvement.

### Chromosome distribution and duplication pattern analysis of the *VQ* genes

The *VQ* genes with chromosomal positions were mapped on the chromosomes of the four *Ipomoea* species with MapChart (ver. 2.30) [65]. The potential duplicated *VQ* genes in the four *Ipomoea* genomes were analyzed with MCScanX software (Multiple Collinearity Scan toolkit X version) [66]. During this stage, the protein sequences of the four *Ipomoea* species were compared against themselves using the BLASTP program (ver. 2.2.28 +) with an E-value of 1e–10. The final output was visualized using the CIRCOS software (ver. 0.66) [67].

### Syntenic analysis *VQ *genes in the four *Ipomoea* genomes

Syntenic block in the genomes of the four *Ipomoea* species was analyzed using MCScan software (Python version) [68] with the default parameters [69, 70]. The gene models were aligned with LAST (ver. 1257), and hits were filtered to locate the best 1:1 syntenic blocks (pairs) and were visualized in the dot-plot script using JCVI package [68].

### Ka/Ks Analysis of duplicated and syntenic *VQ* genes

Both duplicated and syntenic *VQ* gene pairs of the four *Ipomoea* species were selected for the non-synonymous substitution (Ka) to synonymous substitution (Ks) [Ka/Ks] calculation with TBtools (ver. 1.108) [71].

### Promoter analysis of *VQ *genes in the four *Ipomoea* species

The 1,500-bp promoter sequences of the *Ipomoea VQ* genes were summited into PLANT CARE (http://bioinformatics.psb.ugent.be/webtools/plantcare/html/, accessed on 18 March 2021) for identification of the putative *cis*-elements [72].

### Expression profile of *VQ *genes of the four* Ipomoea* genome

For expression profile analysis of the *VQ* genes in the four *Ipomoea* genomes, two bio project datasets (PRJNA511028 for *I. batatas* and PRJDB4356 for *I. nil*) were obtained from the NCBI database. The expressional gene information (fragments per kilobase of exon model per million mapped fragments, FPKM) of *I. trifida* and *I. triloba* was acquired from the Sweetpotato Genomics Resource (http://sweetpotato.uga.edu/). At the same time, two of our in-house transcriptome datasets (unpublished) for sweetpotato stem nematodes and *C. fimbriata* resistance of four sweetpotato cultivars or lines (sweetpotato stem nematodes susceptible cultivar, “Tengfei,” sweetpotato stem nematodes resistant line, “JK20,” *C. fimbriata* susceptible cultivar, “Santiandao” and *C. fimbriata* resistant line, “JK274”) were used. After removing the low-quality reads and adaptor trimming by TrimGalore software (ver. 0.6.4) (trimming parameter: –q 25 –phred33 –length 36 -e 0.1), the clean ribonucleic acid (RNA)-Seq reads were aligned to the sweetpotato reference genome sequences via Hisat2 (ver. 2.0.4). After that, aligned read counting has been done using SAMtools software (ver. 1.11) [73]. Then the obtained read counts were imported into DEseq2 (ver. 1.30.1) for the analysis of DEGs. In each comparing case, reads were treated as DEGs if |log2FC|> 1 and FDR ≤ 5%. Thus, the mean log2FC value for each DEG was calculated. The heat map was constructed to visualize the distribution of the expression level of genes using the reads per kilobase per million (RPKM) value in MeV software [74].

### RNA isolation and quantitative qRT-PCR analysis

The cultivars, Tengfei (susceptible cultivar) and JK20 (resistant line) were inoculated with stem nematodes [75] and the cultivars, Santiandao (susceptible cultivar) and JK274 (resistant line) were inoculated with *C. fimbriata* [76]. Samples were collected at seven-time points (0 h, 6 h, 12 h, 1 day, 2 days, 4 days, and 6 days) after the injection. Root samples without injection were used as a control or mock. Xu32 (susceptible cultivar) and JK328 (resistant line) were selected for cold, salt and drought stress treatments. The cuttings about 25 cm in length from 6-week-old of them grown in a field were cultured in the Hoagland solution [77] for three days to survive: for cold stress treatment, the cuttings were then placed on 28 °C (control) and 16 °C (cold stress), respectively; for salt stress treatment, the cuttings were cultured in the Hoagland solution with 0 and 86 mM NaCl, respectively; for drought stress treatments, the cuttings were cultured in the Hoagland solution with 0 and 30% PEG6000. Samples were collected at seven-time points (0, 2, 4, 6, 12, 24, and 48 h) after the treatments. Then the total RNA of the samples was isolated using RNAprep Pure Plant Kit (Tiangen Biotech, Beijing, China) and first-strand cDNA was synthesized by Quantscript Reverse Transcriptase Kit (Tiangen Biotech, Beijing, China). The sweetpotato β-actin gene (Genbank AY905538) was used as a control and to normalize the relative quantities of the three individual targeted DEGs based on its consistency across the different time points of each treatment of stem nematode and *C. fimbriata*. Three biological replicates were performed at each time point, and the gene expression changes were calculated using the 2^–Δ Δ Ct^ method for each sample [78]. The qRT-PCR was performed as described previously [79] using the generated primers (Additional file 8: Table S8) through Primer-BLAST software [80].

## Supplementary Information

**Additional file 1: Table S1.** VQ gene family information of sweetpotato, I. trifida, I. triloba and I. nil.file.

**Additional file 2: Table S2.** Duplication pattern in sweetpotato, I. trifida, I. triloba and I. nil.

**Additional file 3: Table S3.** VQ orthologous gene pairs in the four Ipomoea species.

**Additional file 4: Table S4.** VQ orthologous groups among the four Ipomoea species.

**Additional file 5: Table S5.** Ka and Ks of VQ genes within or between sweetpotato, I. trifida, I. triloba and I. nil.

**Additional file 6: Table S6.** Statistics of stress-related regulatory elements in the eight phylogenetic groups.

**Additional file 7: Table S7.** The differentially expressed VQ genes response to sweetpotato biotic and abiotic stresses.

**Additional file 8: Table S8.** Primers used in qRT-PCR.

## Data Availability

The datasets generated or analyzed in this study are included in this paper and its supplementary information files. The open RNA-seq datasets (accession numbers PRJNA511028, PRJDB4356, PRJNA631585, PRJNA413661 and PRJNA341328) used and analyzed in this study are available in the NCBI database.

## Abbreviations

*VQ*: Valine glutamine
DEGs: Differentially expressed genes
qRT-PCR: Quantitative reverse-transcription polymerase chain reaction
TFs: Transcription factors
TCs: Transcription complexes
Ka: Non-synonymous substitution
Ks: Synonymous substitution
Ka/Ks: Non-synonymous substitution to synonymous substitution
